# Cancer Incidence among Adolescents and Young Adults in Urban Shanghai, 1973–2005

**DOI:** 10.1371/journal.pone.0042607

**Published:** 2012-08-03

**Authors:** Qi-Jun Wu, Emily Vogtmann, Wei Zhang, Li Xie, Wan-Shui Yang, Yu-Ting Tan, Jing Gao, Yong-Bing Xiang

**Affiliations:** 1 Department of Epidemiology, Shanghai Cancer Institute, Renji Hospital, Shanghai Jiaotong University School of Medicine, Shanghai, China; 2 State Key Laboratory of Oncogene and Related Genes, Shanghai Cancer Institute, Renji Hospital, Shanghai Jiaotong University School of Medicine, Shanghai, China; 3 Department of Epidemiology, University of Alabama at Birmingham, Birmingham, Alabama, United States of America; The University of Texas M. D. Anderson Cancer Center, United States of America

## Abstract

**Background:**

Lack of cancer incidence information for adolescents and young adults led us to describe incidence trends within the young population of 15 to 49 year-olds in urban Shanghai between 1973 and 2005.

**Methods:**

During 1973 to 2005, data on 43,009 (45.8%) male and 50,828 (54.2%) female cancer cases aged 15–49 years from the Shanghai Cancer Registry were analyzed. Five-year age-specific rates, world age-standardized rates, percent change (PC), and annual percent change (APC) were calculated using annual data on population size and its estimated age structure.

**Results:**

During the 33-year study period, overall cancer incidence of adolescents and young adults among males marginally decreased by 0.5% per year (P<0.05). However, overall cancer incidence for females slightly increased by 0.8% per year (P<0.05). The leading cancer for males in rank were liver, stomach, lung, colorectal, and nasopharyngeal cancers and for females were breast, stomach, colorectal, thyroid, and ovarian cancers. Among specific sites, incidence rates significantly decreased for cancers of the esophagus, stomach, and liver in both sexes. In contrast, incidence rates significantly increased for kidney cancers, non-Hodgkin lymphoma, and brain and nervous system tumors in both sexes and increased for breast and ovarian cancers among females.

**Conclusions:**

Overall cancer incidence rates of adolescents and young adults decreased in males whereas they increased in females. Our findings suggest the importance of further epidemiology and etiologic studies to further elucidate factors contributing to the cancer incidence trends of adolescents and young adults in China.

## Introduction

It has been estimated that there are approximately 350,000 new cancer diagnoses annually in the age group of 15 to 29 years and an additional 650,000 cases in the age group of 30 to 39 years worldwide [1]. Because the majority of these incident cases occur in low-income countries, a large proportion of actual cases are likely not diagnosed or those diagnosed may not receive adequate therapy [1].

Cancer incidence patterns of adolescents and young adults in developed countries have been well-studied [2]–[5], but less is known in developing countries, especially using population-based registration data [6]. Unlike children [7] and the elderly population [8], to our knowledge, there has not been any publication on cancer incidence among adolescents and young adults in Shanghai or within any part of China; and there have been only a few reports from other developing countries [6]. Information on cancer incidence patterns will not only provide a basis for additional etiological research but also help identify opportunities and set priorities for cancer control interventions [9], [10]. Therefore, this study examined cancer incidence trends among adolescents and young adults aged 15 to 49 years-old for selected cancers by sex using data from the population-based Shanghai Cancer Registry.

## Materials and Methods

### Cancer registry and patients

The Shanghai Cancer Registry, an associate member of the International Agency for Research on Cancer (IARC), has required all medical facilities within Shanghai to report newly diagnosed cancer cases and cases of non-malignant tumours of the central nervous system since 1972. Additionally, this cancer registry has been included in the most recent volumes of Cancer Incidence in Five Continents and uses standard procedures for collection, processing and reporting of data. The introduction of Shanghai Cancer Registry and detail of registration practices have been described elsewhere previously [7], [11], [12].

During the 33-year period included in our study, changes in the *International Classification of Diseases* (ICD) catalog occurred. The anatomic sites of cancer cases were coded using both the ICD-9 and ICD-10 from 1998 to 2001 and using only ICD-10 since 2002. The histology and behavior of the tumor was coded using *International Classification of Diseases for Oncology, Second Edition* (ICD-O-2) topography (site) and morphology codes [13] since 2002. Due to the changes in the registry catalogs during the period, all the cancer diagnosis codes were converted into ICD-9.

Numerical indices of data quality were calculated for the diagnostic criteria of histological verification (HV) and death certificate only (DCO) [14]. HV was divided into two parts, histological detection and cytological or biochemical detection (Table 1). Histological detection includes diagnoses based on histology of primary, metastasis, and autopsy. Cytological or biochemical detection includes exfoliative cytology and haematological examination of peripheral blood. We also included the proportion of patients diagnosed using surgery or medical imaging (including B ultrasound, X-ray, computed tomography, etc.), and clinical deduction. In our study, these indices were calculated separately for eleven 3-year time intervals from 1973–1975 to 2003–2005.

**Table 1:** Proportion (%) of diagnostic evidence for all incident cancer cases among adolescents and young adults in urban Shanghai, China (1973–2005).

		Diagnostic evidence				
Period	No. of	Histological	Cytological or	Surgery or	Clinical	Death
	cases	detection	biochemical detection	medical imaging	deduction	certificate
1973–1975	7877	59.8	0.6	21.9	3.2	14.5
1976–1978	7230	59.5	0.6	19.6	3.0	17.3
1979–1981	6525	65.0	1.1	17.4	2.9	13.6
1982–1984	6346	66.2	2.0	14.1	2.8	14.6
1985–1987	6912	69.4	1.6	14.4	2.8	11.8
1988–1990	8296	74.3	1.4	18.0	4.2	2.1
1991–1993	8999	73.2	2.7	20.8	3.2	0.1
1994–1996	8994	70.2	4.2	22.5	3.0	0.1
1997–1999	10187	72.7	4.3	21.2	1.7	0.1
2000–2002	11113	76.9	4.8	17.0	1.0	0.3
2003–2005	11358	79.6	2.7	14.3	1.8	1.6
Total	93837	71.1	2.6	18.4	2.5	5.4

There is growing acceptance that adolescence encompasses the age range from 15 to 19 years [15] , but there is much less agreement on the upper age limit for young adults [1], [9], [16]–[18]. We used the widest age range defined by the published research [9] and included people aged 15 to 49 years-old as the adolescent and young adult population in our study. Population estimates were based on 9 periodic national or Shanghai city censuses (1973, 1979, 1982, 1985, 1990, 1992, 1996, 2000, 2005), with age- and sex-specific annual estimates derived by linear interpolation for the remaining years [11]. In this study, all common incident cancers and those with large sex differences that were registered by the Shanghai Cancer Registry between 1973 and 2005 among patients from 15 to 49 years old were included (Table 2).

**Table 2:** Cancer incidence for adolescents and young adults in Shanghai by sex, 1973–1975 and 2003–2005.

						Percent			
Cancer site (ICD-9)	Sex	1973–1975		2003–2005		Change§	APC‡	*P*	95%CI
						(%)	(%)	value*	
		Case	Rate†	Case	Rate†				
Nasopharynx (147)	Male	162	3.3	204	2.9	−12.1	−0.5	0.1883	−1.3, 0.3
	Female	101	2.1	72	1.1	−47.6	−2.0	<0.0001	−2.6, −1.4
Esophagus (150)	Male	232	4.5	121	1.3	−71.1	−3.6	<0.0001	−5.0, −2.2
	Female	82	1.6	4	0.1	−93.8	−8.1	<0.0001	−9.6, −6.6
Stomach (151)	Male	663	13.0	538	6.6	−49.2	−2.1	<0.0001	−2.5, −1.7
	Female	435	8.9	412	5.6	−37.1	−1.1	<0.0001	−1.5, −0.7
Colon and rectum	Male	259	5.4	486	6.2	14.8	0.1	0.8567	−0.4, 0.4
(153, 154)	Female	312	6.6	416	5.5	−16.6	−0.3	0.2700	−0.9, 0.3
Liver (155)	Male	956	18.9	852	10.5	−44.4	−1.7	<0.0001	−2.1, −1.3
	Female	212	4.1	132	1.8	−56.1	−2.1	<0.0001	−2.7, −1.5
Lung and bronchus	Male	373	7.3	562	6.6	−9.6	0.1	0.8439	−0.5, 0.7
(162)	Female	195	3.9	290	3.7	−5.1	0.1	0.6015	−0.3, 0.5
Bone and joint (170)	Male	57	1.1	59	1.0	−9.1	−0.5	0.2457	−1.5, 0.5
	Female	52	1.1	38	0.8	−27.3	−1.1	0.0213	−1.9, −0.3
Connective and soft	Male	43	0.9	48	0.8	−11.1	0.8	0.2617	−0.6, 2.3
tissue (171)	Female	38	0.8	49	0.8	1.0	0.4	0.4416	−0.6, 1.4
Breast (174)	Female	656	13.3	2270	29.9	124.8	2.9	<0.0001	2.5, 3.4
Cervix (180)	Female	580	11.1	458	7.7	−30.6	−1.9	0.1379	−4.3, 0.5
Ovary (183)	Female	177	3.7	414	6.1	64.9	2.1	<0.0001	1.7, 2.5
Testis (186)	Male	23	0.5	50	1.0	100.0	0.9	0.1188	−0.1, 1.9
Kidney (189)	Male	17	0.3	218	2.9	866.7	8.2	<0.0001	7.1, 9.3
	Female	24	0.5	107	1.7	240.0	4.8	<0.0001	3.5, 6.1
Brain and nervous	Male	141	2.9	215	3.3	13.8	1.1	0.0005	0.5, 1.7
(191, 192)	Female	110	2.3	264	4.3	87.0	2.0	<0.0001	1.6, 2.4
Thyroid (193)	Male	92	1.9	155	2.4	26.3	0.8	0.2315	−0.6, 2.3
	Female	350	7.6	544	9.2	21.1	1.0	0.1366	−0.2, 2.2
Hodgkin disease (201)	Male	38	0.7	19	0.4	−42.9	−0.4	0.6438	−2.2, 1.4
	Female	23	0.5	12	0.3	−40.0	−1.1	0.1976	−2.9, 0.7
Non- Hodgkin	Male	97	2.0	168	2.6	30.0	0.7	0.0035	0.3, 1.1
lymphoma (200, 202)	Female	70	1.5	141	2.3	53.3	1.6	0.0005	0.8, 2.4
Leukemia (204–208)	Male	167	3.3	180	3.2	−3.0	−0.4	0.1085	−0.8, 0.0
	Female	150	3.1	144	2.6	−16.1	−1.1	0.0048	−1.9, −0.3
Lymphoid Leukemia	Male	30	0.6	41	0.8	33.3	0.7	0.2569	−0.5, 1.9
(204)	Female	21	0.4	27	0.5	25.0	0.4	0.6155	−1.2, 2.1
Myeloid Leukemia	Male	88	1.7	90	1.5	−11.8	−1.0	0.0080	−1.6, −0.4
(205)	Female	73	1.6	69	1.2	−25.0	−1.4	0.0042	−2.4, −0.4
Other Leukemia	Male	49	0.9	49	0.8	−11.1	−0.3	0.6318	−1.3, 0.7
(206–208)	Female	56	1.2	48	0.8	−33.3	−1.5	0.0052	−2.5, −0.5
All cancer	Male	3776	75.1	4680	62.1	−17.3	−0.5	0.0036	−0.9, −0.1
	Female	4101	83.5	6678	96.7	15.8	0.8	<0.0001	0.4, 1.2

ICD-9, *International Classification of Diseases*, Ninth Revision; APC, annual percent change; CI, confidence interval.

† Age-standardized rate to the World Standard Population.

* P value is calculated for the APC.

‡ APC was calculated based on age-standardized (world population, per 100,000) incidence rate.

§ Percent change between 1973 and 2005 was calculated by the age-standardized rate (world population).

### Statistical analysis

Incidence rates were calculated for eleven 3-year time intervals from 1973–1975 to 2003–2005. Age-standardized rates were estimated by the direct method, using the World Standard Population and expressed as per 100,000 people [19]. For each sex, overall age-standardized incidence rates were computed, and age-specific rates as well. The male to female incidence rate ratio (IRR) was calculated by the age-standardized incidence rates. The annual percentage change (APC) for incidence rates was used to quantify the time trends [20]. A regression line was fitted to the natural logarithm of the rates, weighted by the number of cases, i.e. 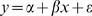, where y = ln (rate) and x = calendar year, and then the APC was calculated as *100×(e^β^-1)*. The 95% confidence interval (CI) of the APC was calculated by the methods for population-based cancer statistics recommended by the National Cancer Institute (21). All analyses were conducted using SPSS for Windows (version 11·5, SPSS Inc, Chicago, IL, USA). All statistical tests were two-sided, and P-values less than 0.05 were considered statistically significant.

## Results

### Overall cancers

Between 1973 and 2005, a total of 43,009 (45.8%) male and 50,828 (54.2%) female cancer cases aged 15 to 49 years-old were registered by the Shanghai Cancer Registry. During this period, 73.7% of records were based on HV and 5.4% of diagnoses were based on DCO. During the 33-year period, there was a sharp reduction in the percentage of records based on DCO cases, from 14.5% to 1.6% (Table 1).

**Figure 1:**
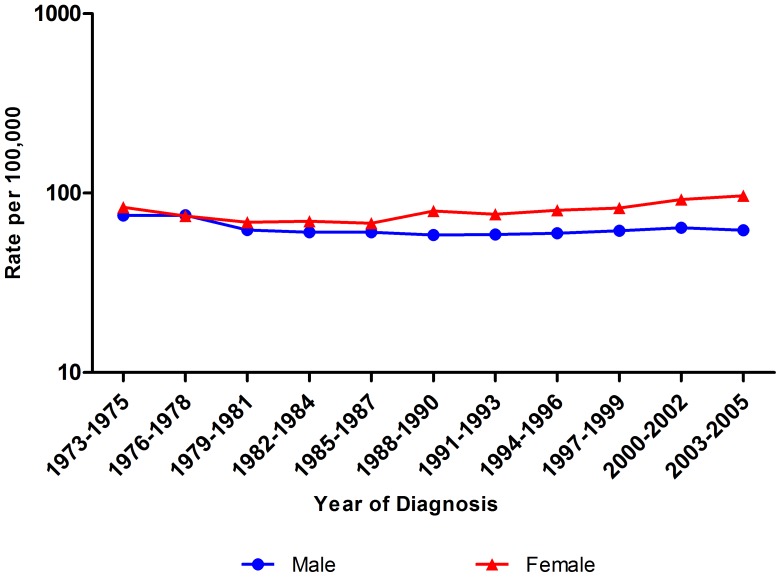
Trends in overall cancer incidence rates (age standardized to the World Standard Population, per 100,000) among adolescents and young adults, 1973–2005.

The counts of cases and age-standardized incidence rates by cancer site are presented in Table 2 for the periods 1973–1975 and 2003–2005. For all cancers, the combined rates in males decreased by 17.3% from 75.1 to 62.1 per 100,000, or 0.5% per year, whereas the rates in females increased by 15.8% from 83.5 to 96.7 per 100,000, or 0.8% per year (Table 2, Figure 1). Between 1973 and 1975, 7,877 cases of cancer were diagnosed in urban Shanghai. In males, the top five cancer sites were cancer of the liver, stomach, lung, colorectum, and esophagus which accounted for 65.8% of all male cancer cases during this period. During the same period, in females, the top five cancer sites were of the breast, cervix, stomach, thyroid, and colorectum which accounted for 62.4% of all female cases. During the most recent time period, 2003–2005, 11,358 cases of cancer were diagnosed in urban Shanghai. Liver, lung, stomach, colorectal, and brain and nervous system tumors were the five leading sites of cancer among males, which accounted for 56.7% of all male cancer cases. For females, cancers of the breast, thyroid, cervix, ovary and stomach were the most common sites. These five leading cancers accounted for 63.7% of all cases diagnosed among females during the period of 2003–2005 (Table 2).

### Digestive system cancers

**Figure 2:**
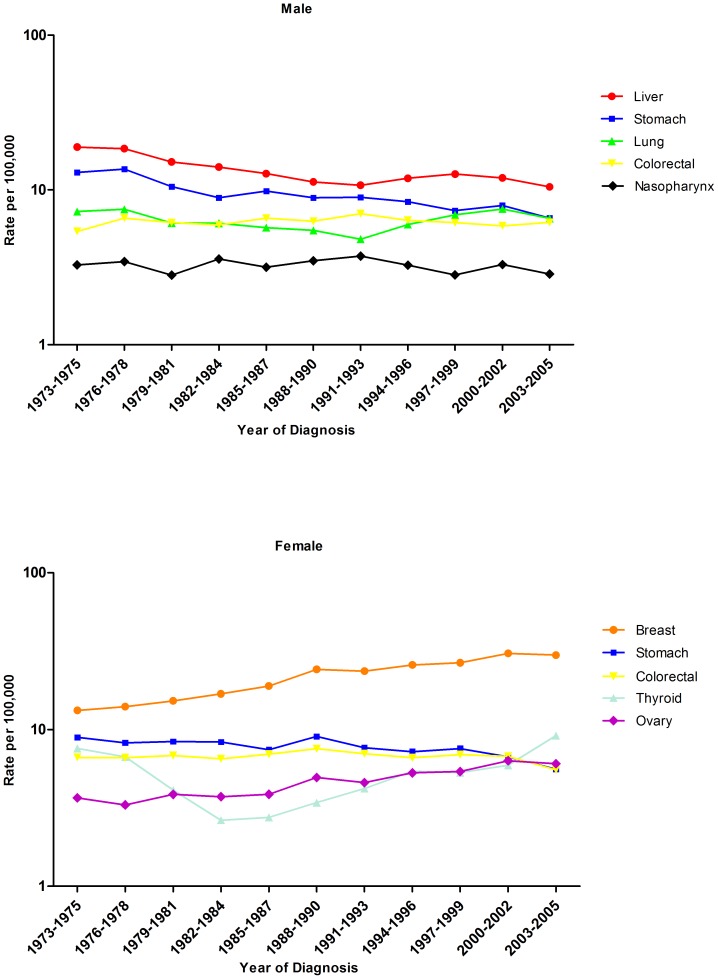
Trends in incidence rates (age standardized to the World Standard Population, per 100,000) at the top five cancer sites by sex among adolescents and young adults, 1973–2005.

Over the study period, stomach cancer was the most commonly diagnosed cancer, accounting for 12.2% of all cancers in adolescents and young adults overall. Between 1973–1975 and 2003–2005, the incidence rates of stomach cancer decreased by 49.2% in males and 37.1% in females (Table 2, Figure 2). In males, stomach cancer rarely occurred before the age of 25, but the incidence increased rapidly after the age of 30, while in females, the incidence of stomach cancer increased steadily after the age of 25 (Figure S1).

Liver cancer was the second (11.6%) most commonly diagnosed cancer among both sexes and the first (21.1%) among males. The incidence rates for liver cancer declined more rapidly over time for females than for males, 56.1% vs. 44.4% (Table 2). The incidence rates of liver cancer increased sharply after the age of 30 for both sexes (Figure S1). And liver cancer was observed to be more common among males than females, with a male to female IRR of 4.7.

Cancer of the colon and rectum accounted for 9.3% of all cancers diagnosed in adolescents and young adults. Although the rate of colorectal cancer remained relatively stable over time and showed no significant changes among both sexes, the age-standardized incidence of colorectal cancer increased uniformly with age in all four time-periods for both sexes (Figure S1).

Although esophageal cancer accounted for only 1.5% of all cancers, esophageal cancer decreased 68.9% among males and 71.1% among females from 1973 to 2005. After the age of 35, irrespective of sex, the incidence rates for cancer of esophagus increased sharply (Figure S1). Second to liver cancer, esophageal cancer was more common among males than females with a male to female IRR of 2.4.

### Female breast, ovarian and cervical cancer

Over the 33-year study period, breast cancer accounted for 29.0% of all cancers diagnosed in females. Breast cancer incidence rates increased substantially over this time period, rising 124.8% from 13.3 per 100,000 in 1973–1975 to 29.9 per 100,000 in 2003–2005, or 2.9% per year (Table 2, Figure 2). The incidence of breast cancer increased progressively with age, especially after the age of 35. Breast cancer rates were highest among the period 1997–2005 than any other period (Figure S2).

Ovarian cancer accounted for 5.8% of all cancers diagnosed in females. Cancer of the ovary increased by 64.9% over the study period, becoming one of the leading cancers among females in recent years (Table 2). Over all time periods, after the age of 35, the incidence rate of ovarian cancer increased steadily with age (Figure S2).

Cervical cancer accounted for 3.7% of all cancers diagnosed in females. There was no statistically significant change over time for cervical cancer with an APC of −1.9% (95% CI: −4.3, 0.5). During the period of 1973–1980, the incidence rates increased significantly with age, especially after the age of 30. In contrast, the age-specific incidence rates during the other three time periods were much lower than the period of 1973–1980 (Figure S2).

### Lung cancer

During this 33-year period, lung cancer, one of the most common cancers in Shanghai, accounted for 10.2% of all cancers diagnosed in males and 4.8% in females, but the APC change was not statistically significant and similar among both sexes over time (Table 2, Figure 2). Cases of lung cancer were rare before the age of 25, but increased rapidly thereafter (Figure S2).

### Kidney cancer

Kidney cancer accounted for only 1.3% of all cancer diagnosed among adolescents and young adults during the 33 year study period and less than 0.6% during the period of 1973–1975. The age-standardized rates increased 8.2% per year in males and 4.8% in females from 1973 to 2005 (Table 2). During the period of 1973–1975, kidney cancer was observed to be more common among females than among males, with the male to female IRR of 0.6. However, in 2003–2005, kidney cancer was found to be more common among males with an IRR of 1.7.

### Leukemia

Leukemia accounted for 3.9% of all cancers diagnosed in adolescents and young adults overall and 4.7% of cancer diagnosed in males and 3.2% in females. For adolescents and young adults, myeloid leukemia was the most common type, accounting for 49.2% of the leukemia cases in males and 46.9% in females. With the exception of lymphoid leukemia in females, all of the rates of leukemia in males or females decreased over time with APCs ranging from −1.5% to −0.3%.

### Other cancers

Cancer of the thyroid accounted for 4.4% of all cancers diagnosed in adolescents and young adults and 2.1% of cancer diagnosed in males and 6.4% in females. The incidence rate of thyroid cancer increased with an APC of 0.8% (95% CI: −0.6, 2.3) and an APC of 1.0% (95% CI: −0.2, 2.2) in males and females, respectively. Thyroid cancer was more common among females than among males, with a male to female IRR of 0.3. The rates of nasopharyngeal cancer significantly decreased during the study period with an APC of −2.0% (95%CI: −2.6, −1.4) in females but showed non-significant decrease in males (Table 2, Figure 2). Although low incidence rates of non-Hodgkin disease and brain and nervous system tumors were observed, the incidence rates for these cancers showed statistically significant increases for both sexes.

## Discussion

This study is the first to describe the secular cancer incidence trends for adolescents and young adults in China. Our findings indicate an increase in cancer incidence among females and a slight decrease among males over the 33 year period in this study. Within specific cancer sites, incidence rates significantly decreased for cancers of the esophagus, stomach and liver and for myeloid leukemia in both sexes; incidence rates significantly decreased for leukemia, bone and joints cancer, and nasopharyngeal cancer only in females. In contrast, incidence rates significantly increased over time for kidney cancers, non-Hodgkin lymphoma, and brain and nervous system tumors in both sexes, with increases in incidence rates for breast and ovarian cancers in females only. Our study was unique compared to the previous literature due to the selected age range of study, 15 to 49 years-old, which was selected to address the gaps in the literature about this population in developing countries.

Compared to many developed countries which began their population-based registries in the 1940s, cancer registries in China started collecting information on cancer incidence in 1972 and were vulnerable to several common technical problems that could jeopardize the quality of the data collected [5]. Compared with the early years of the registry, higher proportions of HV, cytological or biochemical detection and lower proportions of DCO were observed recently. One of the possible explanations for such differences could be the improvement of quality control and data collection in the cancer registry and increased access to modern diagnostic procedures [21]. During the approximate thirty years of registry operation, the cancer registry, as well as the infrastructural development of China, was experiencing a transition. Supported by the Chinese government and the increasing cooperation with international associations such as IARC, the cancer registry and quality control procedures have improved substantially [22].

Compared with previously published cancer incidence data among children aged 0–14 years old and elderly people aged over 60 years old in Shanghai [7], we identified several differences within our population. No overall significant cancer incidence trends were observed for children and elderly people during this time period which is in contrast with the significant decreases among males and increases among females in young population [7]. Within specific sites, liver cancer accounted for a higher proportion of all cancers among the adolescents and young adults than children (11.6% vs. 3.1%) [7]. Colorectal cancer and Hodgkin lymphoma significantly increased within the elderly population but showed no significant changes over time among adolescents and young adults [8]. Among females, the decrease in cervical cancer (APC: −8.5% vs. −1.9%) was much more dramatic within the elderly population than among adolescents and young adults [8]. In addition, the common type of leukemia changed from lymphocytic sub-type in childhood [7] to myeloid in adolescents and young adults, myeloid sub-type accounting for 21.3% (children) vs. 49.2% (adolescents and young adults) of the leukemia cases in males and 20.9% vs. 46.9% in females [7].

Our study revealed that stomach cancer ranked second in both sexes over the study period in Shanghai, China. With the rediscovery of Helicobacter pylori (*H. pylori*) in the early 1980s by Warren and Marshall, epidemiology research [23], [24] demonstrated that chronic bacterial infection with *H. pylori* was the strongest risk factor for stomach cancer. Due to these findings, *H. pylori* infection was established as a risk factor for stomach cancer by IARC [25]. The cause of the significant decrease in incidence rates of stomach cancer within this population is not fully understood, but it is believed that the decreased reliance on salted and preserved foods after the invention of refrigeration, the increased availability of fresh fruits and vegetables, improved hygiene and the use of antibiotics may have caused a reduction in chronic *H. pylori* infection which in turn decreased the incidence of stomach cancer [26]. Since research on cigarette smoking and alcohol drinking in relation to stomach cancer is lacking within this population, we cannot conclude whether these risk factors may have played a role in the trend of stomach cancer among adolescents and young adults.

The reduction in the incidence of liver cancer among both sexes in our study is likely related to routine vaccination for hepatitis B as previously suggested [27]. Since the 1980s, Shanghai has implemented infant hepatitis B vaccination as standard of primary care according to Chinese health ministry recommendations [28]. The significant decrease in liver cancer in our study may also be related to the general improvements in living conditions, quality of drinking water, and diet such as the widespread use of refrigeration, improvements in food storage and transportation, and reduced exposure to aflatoxin [29], [30]. The cause for the higher incidence rate of liver cancer among males compared to females is not completely understood, but may be partly explained by the sex-specific prevalence of risk factors like alcohol drinking, cigarette smoking and infection with hepatitis B virus (HBV) and hepatitis C virus (HCV) [29].

Previous epidemiological research showed that the incidence rates of colorectal cancer start to increase after the age of 35 and had a rapid increase after the age of 50, when more than 90% of all colorectal cancers developed [31]. These results support our findings that the incidence rates of colorectal cancer were lower among the adolescent and young adult population compared to those seen in the elderly population. Within adolescents and young adults, family history of colorectal cancer is one of the few established risk factors for the disease [32]. It is still unclear whether dietary and lifestyle factors associated with Westernization and early cancer screening such as fecal occult blood testing and sigmoidoscopy affect the trend of colorectal cancer among adolescents and young adults.

Although a low incidence rate of esophageal cancer and small proportion of cases were observed, the APC of esophageal cancer showed a significant decrease in both sexes. The striking male predominance of esophageal cancer within our study was also observed in many other parts of the world [33]. Some research, therefore, has proposed that the difference in esophageal cancer incidence between males and females might be explained by different exposures to sex hormones, with possible protective effects of estrogen and/or progesterone and a harmful effect of testosterone [34], [35]. On the other hand, similar to other digestive system cancers, the general improvements in living conditions and diet also may have contributed to this significant decline in the incidence of esophageal cancer [36].

As the largest contributor, breast cancer was responsible for major changes of the increasing trend in females. With the exception for the family history and genetic predisposition [37]–[39], the prevalence of risk factors for female breast cancer have been changing dramatically in adolescents and young adults with the rapid economic development in Shanghai over the past three decades. For example, there have been substantial increases in the proportion of dietary calories from fat [40] and a decline in physical activity among urban residents of China which has led to rapid increases in the prevalence of obesity [39], [41]. Additionally, changes in reproductive factors e.g. age at menarche, parity, age at first full-term pregnancy, lactation, etc and hormonal level also may have contributed to the significant increase in breast cancer incidence [42]–[45].

For leukemia, the predominant leukemia sub-type is lymphocytic in children and myeloid in adults [7], [46]–[49]. The reasons for the change in the histological type of leukemia with increasing age are not well-known. With the exception of exposure to ionizing radiation and specific genetic syndromes, little is known about the risk factors for leukemia [50], [51]. Similarities in the risk factors for, but differences in the tumor biology of leukemia among children and adolescents and young adults may suggest that the etiologic factors or host susceptibility may not be the same [52]–[54].

For kidney cancer, a significant increase was also observed in the elderly population [7], [8]. However, the increase in kidney cancer was highest in adolescents and young adults. These findings were similar to the result observed from a recent study from the United States [55]. Some research has indicated that cigarette smoking, obesity, and hypertension are the risk factors for kidney cancer [56]–[58]. However, little research is focused on risk factors of kidney cancer among adolescents and young adults and therefore the causes for the increasing trend are not clear.

Our study is not without limitations. First, our data was not all recorded according to the protocols of the ICD-O-2, so topographic and morphologic information were not available for all of the data to analyze the incidence trends by cell type in adolescents and young adults in the Shanghai Cancer Registry. In addition, cancer registration is often incomplete during the initial years of a population-based registry. Although a 7-year ‘run-in period’ is suggested as an ideal for such registries [59], the establishment and development of the cancer registration system in China was later than countries such as the United States or several European nations and so in this study a “run-in period” was not applied.

Despite these limitations, this study is the first descriptive research that focuses on the cancer incidence of adolescents and young adults in China, which has been underrepresented in previous studies. People aged 15 to 49 years-old represent a unique population in cancer occurrence and have a unique distribution of cancers that is different from those in children and the elderly. Monitoring and reporting cancer incidence rates among adolescents and young adults may reflect trends which can help scientists to design new research to study suspected risk factors and the etiology of these cancers. Developing countries, including China, need to conduct more research focusing on cancer occurrence in adolescents and young adults.

## Supporting Information

Figure S1 **Age-specific incidence rates for digestive system cancers by sex among adolescents and young adults in urban Shanghai, 1973–2005.**
(TIF)

Figure S2 **Age-specific incidence rates for lung, breast, and other cancers by sex among adolescents and young adults in urban Shanghai, 1973–2005.**
(TIF)
