# Supplementary figures and images for: Cancer Incidence among Adolescents and Young Adults in Urban Shanghai, 1973–2005
Source: PLoS One. 2012 Aug 3;7(8):e42607. doi: 10.1371/journal.pone.0042607 (PMC3411830; doi:10.1371/journal.pone.0042607)

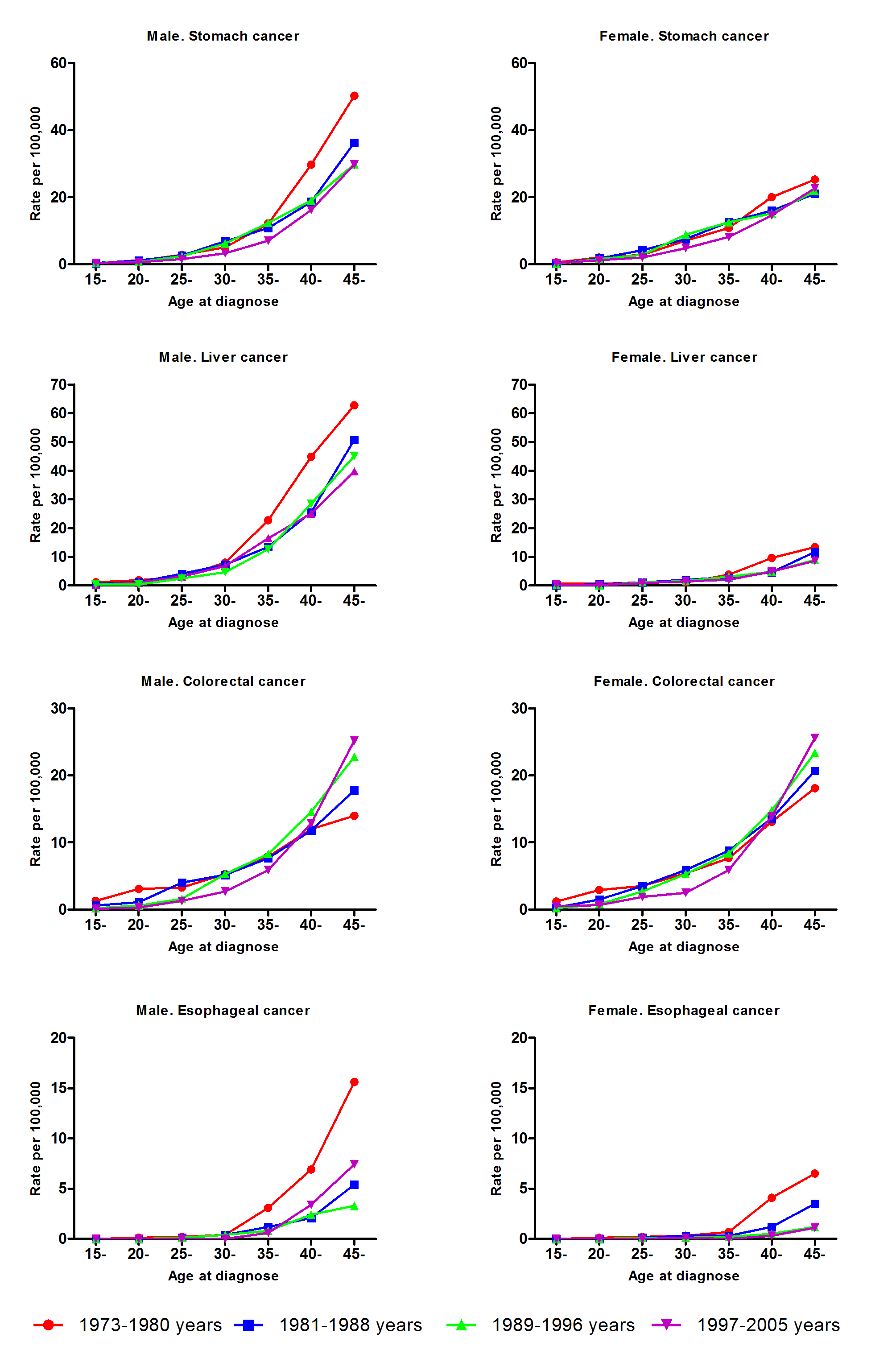

Supplement: Figure S1 — Age-specific incidence rates for digestive system cancers by sex among adolescents and young adults in urban Shanghai, 1973–2005. (TIF) [file pone.0042607.s001.tif]

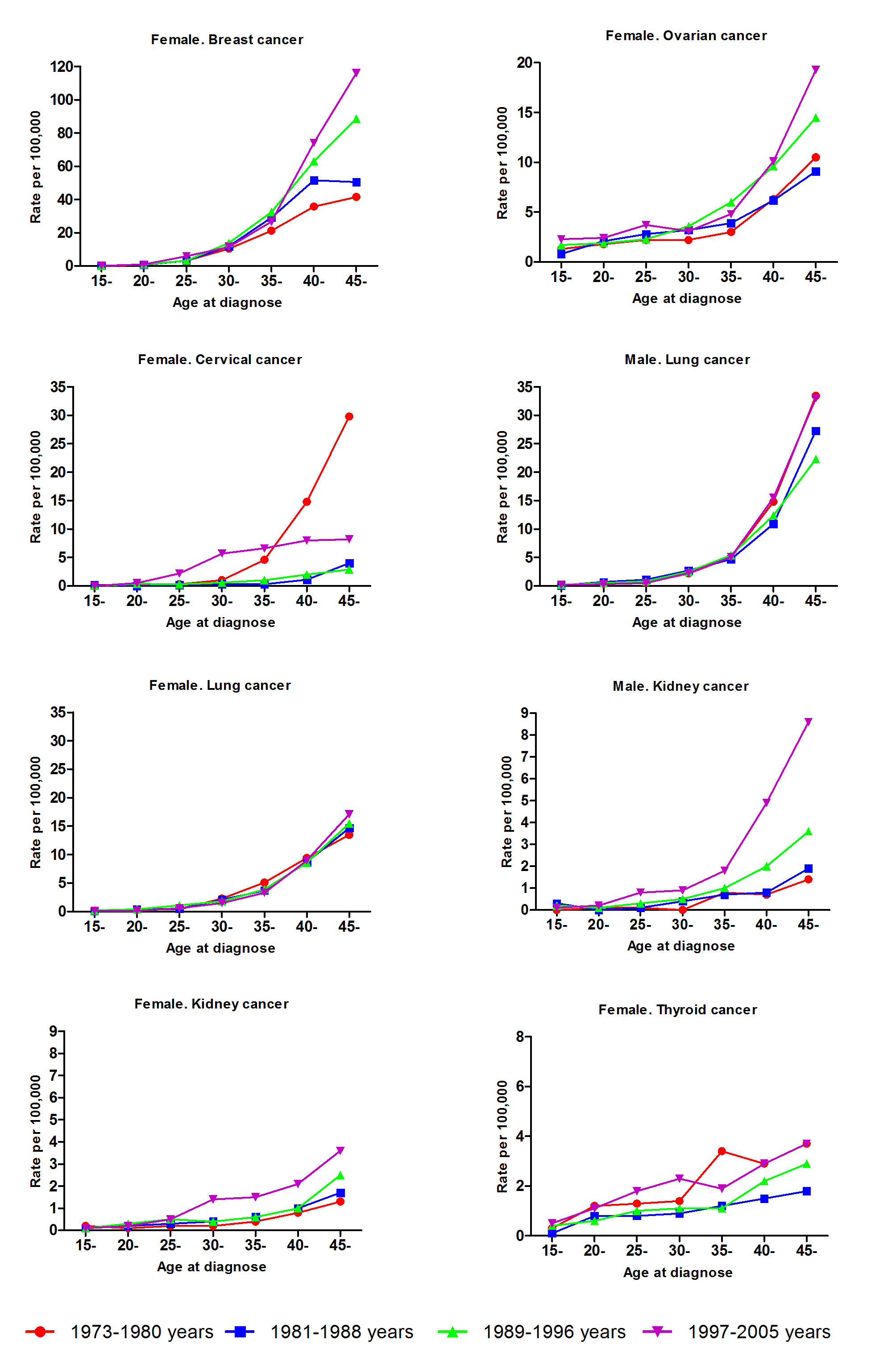

Supplement: Figure S2 — Age-specific incidence rates for lung, breast, and other cancers by sex among adolescents and young adults in urban Shanghai, 1973–2005. (TIF) [file pone.0042607.s002.tif]
